# Digestive manifestations of Covid-19 in children: a retrospective study

**DOI:** 10.4314/ahs.v23i3.22

**Published:** 2023-09

**Authors:** Abderrahmane Jallouli, Karima El Fakiri, Houda Nassih, Rabiy EL Qadiry, Aicha Bourrahouat, Imane Ait Sab, Noureddine Rada, Ghizlane Draiss, Mohammed Bouskraoui

**Affiliations:** 1 Department of Pediatrics, University hospital of Mohammed VI, Marrakesh, Morocco; 2 Faculty of Medicine and Pharmacy, Cadi Ayyad University, Marrakesh, Morocco

**Keywords:** COVID-19, digestive manifestations, children

## Abstract

**Background:**

The world is currently facing a pandemic due to a new species of the Coronaviridae family called SARS-CoV-2, discovered in the city of Wuhan in China in December 2019. The WHO has named the resulting disease COVID-19 (Coronavirus Disease 2019). It has been a global health problem due to its major socio-economic damage. The aim of this study was to show the prevalence of gastrointestinal and hepatic manifestations in symptomatic children with COVID-19.

**Methods:**

We performed a retrospective study, including 36 symptomatic children infected by SARS-CoV-2 hospitalized at the mother and child hospital of university hospital of Mohammed VI, Marrakech in Morocco, over a period of 7 months. Clinical and biological manifestations of the digestive system were evaluated for all patients.

**Results:**

The digestive symptomatology came in second place after the respiratory manifestations. 14 patients (38.89 % of symptomatic patients) in our study had digestive symptoms on admission: 12 (33.33%) presented with diarrhea, 4 (11.11%) had abdominal pain and only one child (2.78%) had vomiting. Aspartate aminotransferase (AST) was elevated in one patient, while alanine transaminase (ALT) was elevated in 6 patients. The prothrombin level was normal in all patients. All patients were discharged with good general condition without morbidity and mortality.

**Conclusion:**

This study concludes with the high prevalence of digestive manifestations of COVID-19 in symptomatic children. There were no severe clinical or biological abnormalities in our study. Digestive manifestations during COVID-19 in children are frequent, which requires the awareness of health professionals

## Introduction

COVID-19 (Coronavirus Disease 2019) is a viral disease secondary to infection with a virus of the coronaviridae family, recently discovered in December 2019 in the city of Wuhan in China, and called SARS-CoV-2 (Severe Acute Respiratory Syndrome CoronaVirus2), due to its phylogenetic proximity to the SARS-CoV responsible for the SARS epidemic in 2003. 1

After its emergence, this infection spread rapidly, and was responsible for devastating consequences, causing millions of infected people and deaths as well as socio-economic damage. Therefore, The World Health Organization (WHO) declared on March 11, 2020 that COVID-19 is considered a global pandemic. 1

The respiratory system is the primary target for SARS-CoV-2. However, the gastrointestinal tract and the liver may also be involved, with clinical and) laboratory signs indicating digestive involvement during COVID-19 in children. 2

The aim of this study was to determine clinical and biological digestive manifestations during SARS-CoV-2 infection in children.

## Materials and methods

We performed a descriptive retrospective study including 78 children, 36 of whom were symptomatic with COVID-19, carried out at the mother and child hospital of university hospital of Mohammed VI, Marrakech in Morocco, over a period of 7 months. (From March 28 to October 28, 2020; 1^st^ wave of COVID-19 in Morocco).

### Inclusion criteria

- Age: less than 15 years old.

- Children hospitalized at the mother and child hospital of university hospital of Mohammed VI in Marrakech.

- Children with gastrointestinal symptoms.

- Symptomatic children with a positive COVID-19 polymerase chain reaction (PCR).

### Exclusion criteria

Patients who previously had no digestive symptoms and who developed them after the start of medical treatment, as they may be due to a drug side effect.

### Epidemiological and clinical data

All the epidemiological (age, sex, underlying medical conditions, contact with confirmed cases) and clinical data of the patients included in this study were evaluated.

### Biological data

The biological parameters evaluated to determine digestive involvement were: Alanine transaminase (ALT), aspartate aminotransferase (AST) and prothrombin level (PT). Normal cut-off level for ALT was 1 to 40 IU / L for infants and 0 to 27 IU / L for children. and for AST was 15 to 60 IU / L for infants and 4 to 50 IU / L for children. Normal PT levels were more than 50% in infants and more than 70% in children.

In order to search for a biological inflammatory syndrome, we also measured the level of C-Reactive Protein (CRP) and procalcitonin. A normal level of CRP was defined by a level less than 10 mg / l, and less than 0.5 mg / l for procalcitonin.

### Evolution

The clinical and biological evolution was evaluated in all the patients included in this study.

## Results

COVID-19 (Coronavirus Disease 2019) is a viral disease secondary to infection by a virus belonging to the coronaviridae family, recently discovered in December 2019 in the city of Wuhan in China, and called SARS-CoV-2 (Severe Acute Respiratory Syndrome CoronaVirus2), due to its phylogenetic proximity to the SARS-CoV responsible for the SARS epidemic in 2003. 1

The respiratory system is the primary target for SARS-CoV-2. However, the gastrointestinal tract and the liver may also be involved, with clinical and laboratory signs indicating digestive involvement during COVID-19 in children. 2

### Epidemiological and clinical features

The mean age of our patients was 6 years and 8 months, with age extremes ranging from 1 month to 15 years, with a sex ratio of 1.12% of the patients in our series had underlying medical conditions (4 cases of asthma, 1 case of severe combined immunodeficiency (SCID), 1 case of diffuse interstitial pneumonitis of unknown origin, 1 case of Trisomy 21 with operated congenital heart disease, 1 case of lymphoproliferative syndrome with autoimmunity,1 case of epilepsy, 1 case of prematurity, 1 case of abdominal tuberculosis). The contact with a confirmed case was present in all patients, 97% was with family members. Digestive symptoms came second after respiratory symptoms, 14 patients (38.89% of symptomatic patients) in our study had digestive symptoms on admission: 12 (33.33%) presented with diarrhea, 4 (11.11%) had abdominal pain and only one child (2.78%) had vomiting (table 1).

**Table 1:** Epidemiological and clinical characteristics of the patients in our series

Variable	Mean	Range
Age (year)	6,67	1 month - 15 years
Interval between symptom onset and admission (day)	4,8	1 – 10
**Variable**	**N**	**%**
Sex		
Boy	18	50%
Girl	18	50%
**Symptomatic patients**	**N= 36**	
**Patients with respiratory symptoms**	**16**	
Cough	8	
Rhinorrhea	7	
Sneeze	2	
Sore throat	2	
**Patients with digestive symptoms**	**14**	
Diarrhea	12	
Abdominal pain	4	
Vomiting	1	
**Patients with neurosensory symptoms**	**6**	
Headache	6	
Anosmia	2	
Ageusia	1	

### Laboratory results

The transaminases (aspartame aminotransferase (AST) and alanine aminotransferase (ALT)) were evaluated in all patients. AST was elevated in one patient (2,7%), while ALT was elevated in 6 patients (16.6% of the population). The prothrombin level (PT) was normal in all patients (mean=95,2%) (table 2).

**Table 2:** Liver function results in children in our series

Variable	Interpretation	N	%
ALT (IU/L)	Normal	30	83.7%
	Abnormal	6	16.6%
AST (IU/L)	Normal	35	97.3%
	Abnormal	1	2.7%
PT (%)	Normal	36	100%
	Abnormal	0	0%

CRP was elevated in 3 patients in our study (8.3 %). All patients had normal procalcitonin levels.

### Evolution

All children were discharged with good general condition without morbidity and mortality.

## Discussion

The pathophysiology of digestive involvement in COVID-19 is unknown. The probable mechanism may be due to the interaction between ACE2 receptors (abundantly present in the small intestine, colon and hepatocytes) and the protein S of SARS-CoV-2. 2,3 Other possible mechanisms include inflammatory responses such as cytokine storm, drug side effects and deregulation of the intestinal flora by immune mechanisms. 4

The mechanisms of diarrhea during infection with SARS-COV-2 are not fully understood, this virus is likely to alter intestinal permeability, responsible for enterocyte malabsorption. ACE-2 receptors are also involved in the absorption of amino acids and in the regulation and homeostasis of the intestinal microbiome. 5 Intestinal microbial dysbiosis has been observed in patients with COVID-19, hence the importance of probiotic treatment.6 The pathogenesis of hepatic injury is multifactorial, and is linked to direct viral involvement, hepatocytic ischemia and hypoxia. Liver damage may be part of systemic failure. 5

The gastrointestinal symptomatology is similar to that seen in adults with COVID-19, 7 they may precede respiratory symptoms. 5 COVID-19 can be revealed by digestive symptoms in children. Children infected with SARS-CoV-2 who report diarrhea and vomiting constitute about 9% of cases, and may reach over 20% in some studies. 5 In our study, 38.89% of patients presented with diarrhea, vomiting, and abdominal pain on admission. Lu et al reported that diarrhea and vomiting were observed in 15 (8.8%) and 11 (6.4%) children respectively in a series of 171 cases. 8 Of the 127 children included in the Giacomet et al study, 28 (22 %) had diarrhea, 12 (9.4 %) presented with vomiting and 8 (6.3 %) had abdominal pain. 9

Some studies have shown that patients with digestive symptoms during SARS-COV-2 infection are more likely to develop severe forms of the disease. 9–11 But to date, the correlation between the presence of digestive signs and severe forms of COVID-19 has not been demonstrated. 7

A mildly elevation of transaminases is common during COVID-19, but severe liver dysfunction is not usual. The incidence of abnormal liver function tests ranges from 14.8% to 78%, manifested primarily by abnormal ALT and AST levels as well as mildly elevated bilirubin levels. 4 Severe COVID-19 patients have higher levels of AST, ALT, and bilirubin. 11 Mildly elevated liver transaminase levels were observed in the series of Dooki et al (27.8% and 38.9% for ALT and AST, respectively), 12 and in the study of Parri et al (8% and 10% for ALT and AST, respectively). 13

This study has some of limitations. First, it was a retrospective study, the data were extracted from the medical files of the patients. Second, the absence of severe and critical cases in our study will not allow the study of digestive manifestations in severe forms of the disease. Third, the sample size was small.

Other similar studies with a larger sample are needed to have more insights into the gastrointestinal manifestations of COVID-19 in children, especially with the emergence of several different strains since the onset of the pandemic.

## Conclusion

This study concludes with the high prevalence of digestive manifestations (diarrhea, abdominal pain and vomiting) of COVID-19 in symptomatic children. There were no severe clinical or biological abnormalities.

Digestive manifestations during COVID-19 in children are frequent, which requires the awareness of health professionals.
